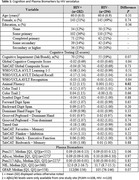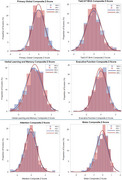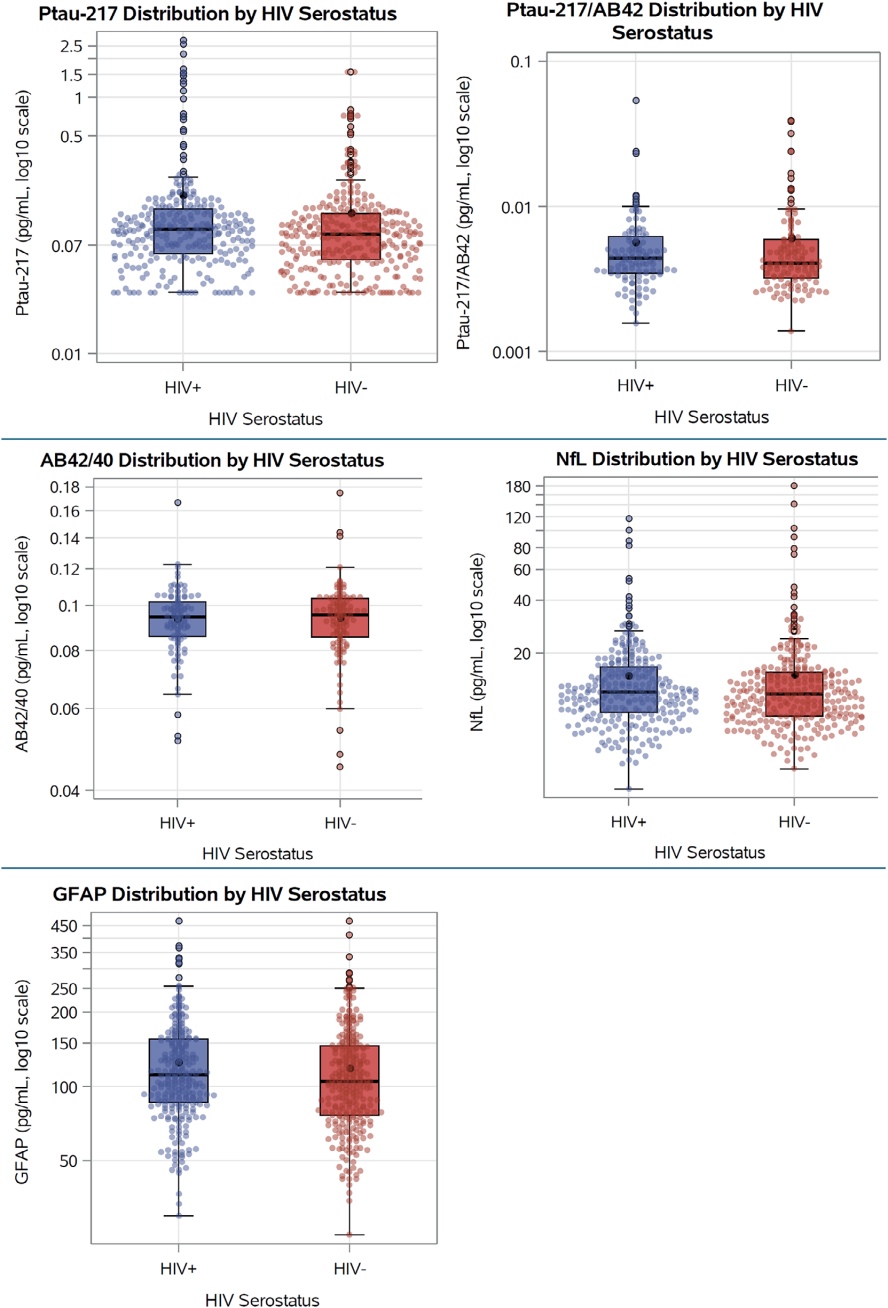# Chronic HIV Infection Association with Cognition and ADRD Plasma Biomarkers in Uganda

**DOI:** 10.1002/alz70857_098870

**Published:** 2025-12-24

**Authors:** Jeremy A. Tanner, Roslyn Valdespino, Tiffany F. Kautz, Godfrey Masette, Gabrielle Hromas, Chen‐Pin Wang, Jayandra Jung Himali, Robert Paul, Noeline Nakasujja, Zahra Reynolds, Flavia Atwine, Edna Tindimwebwa, Meredith Greene, Eliza Passell, Christine S Ritchie, Susanne S Hoeppner, Alexander C Tsai, Seeley Janet, Lawren VandeVrede, Amy Werry, Elena Tsoy, Katherine L. Possin, Sudha Seshadri, Samson Okello, Stephen Asiimwe, Deanna Saylor, Mark J Siedner

**Affiliations:** ^1^ University of Texas Health San Antonio, San Antonio, TX, USA; ^2^ Mbarara University of Science & Technology (MUST), Mbarara, Uganda; ^3^ University of Missouri, St Louis, MO, USA; ^4^ Makerere University, Kampala, Central, Uganda; ^5^ Massachusetts General Hospital (MGH), Boston, MA, USA; ^6^ Kabwohe Clinical Research Center (KCRC), Kabwohe, Uganda; ^7^ Indiana University School of Medicine., Indianapolis, IN, USA; ^8^ London School of Hygiene and Tropical Medicine, London, United Kingdom; ^9^ University of California San Francisco, San Francisco, CA, USA; ^10^ University of North Carolina, Chapel Hill, NC, USA

## Abstract

**Background:**

Sub‐Saharan Africa (sSA) is projected to have the fastest population growth in older adults globally, a >250% increase in Alzheimer's Disease (AD), and rapid growth in older people living with HIV (PLWH) well‐treated on antiretroviral therapies (ARTs) in the coming decades. In the Global North, HIV infection has been associated with neuroinflammation, accelerated brain aging, cognitive impairment, and AD pathology. In sSA, data about the intersections of aging, AD, and HIV are lacking.

**Method:**

The Uganda Aging Cohort Study (UACS) is a prospective cohort study of PLWH well‐treated on ARTs, and age‐ and sex‐matched community‐dwelling people without HIV enrolled with population‐based sampling. Participants completed cognitive testing with the locally normed Ugandan Neuropsychological Battery and tablet‐based Cognitive Assessment Tool (TabCAT). Z‐scores for each test were created using a regression‐based normative approach with adjustment for age, sex, and education. Composite scores were created from mean Z‐scores for global cognition, TabCAT, and each cognitive domain. The presence/absence of cognitive impairment was defined using Jak/Bondi criteria. Plasma *p*‐tau217 and Aβ_42/40_ were measured on the Fujirebio Lumipulse platform. GFAP and NfL were measured on the Quanterix Simoa HD‐X. Chi‐squared and t‐tests were used to compare cognitive performance and natural log‐transformed plasma biomarker levels between groups.

**Result:**

A total of 576 UACS participants (mean age 60±6.5 years, age range 48‐82, 50% female, 51% with less than a primary school education) completed study measures. PLWH had higher scores on animal fluency, but otherwise cognitive performance was comparable between groups including composite scores, cognitive domains, and individual tests (Table 1, Figure 1). There was a trend towards higher frequency of cognitive impairment and elevated *p*‐tau217 among PLWH. There were no statistically significant differences in *p*‐tau217/Aβ_42_, Aβ_42/40,_ GFAP, or NfL by HIV serostatus (Table 1, Figure 2).

**Conclusion:**

Older PLWH well‐treated on ART in Uganda have cognitive performance and plasma ADRD biomarker levels comparable to demographically‐matched people without HIV, consistent with recent studies showing cognitive impairment is less common among PLWH with viral suppression. Ongoing studies will further assess trends in increased cognitive impairment and *p*‐tau217 in PLWH, and evaluate differences in ADRD prevalence and cognitive trajectories in this cohort.